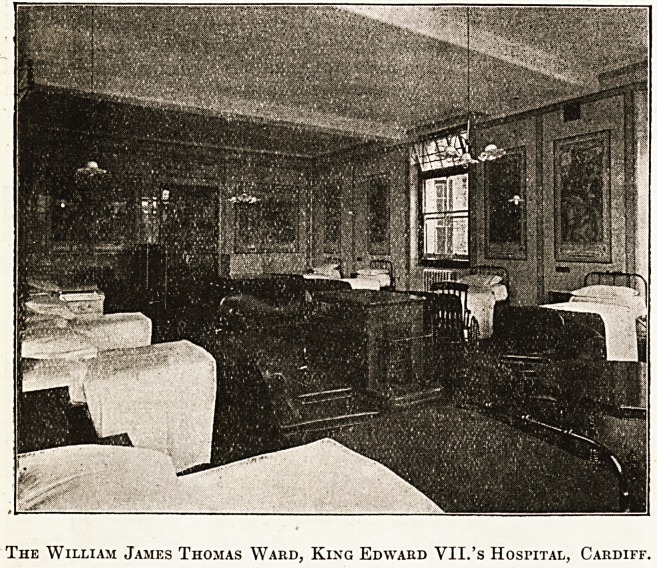# King Edward VII.'s Hospital, Cardiff, Begins a New Era

**Published:** 1912-07-06

**Authors:** 


### THE KING AND QUEEN AT CARDIFF.

### King Edward VII.'s Hospital, Cardiff, Begins a New Era.

THE longest visit ever paid by Royalty to Cardiff was fitly completed on Friday last week, when the King and Queen, *en route* to the railway station, visited the King Edward VII.'s Hospital and were received by the Lord Mayor of Cardiff, Alderman Sir John W. Courtis. The following had the honour of being presented to their Majesties by the Lord Mayor, and afterwards conducted the King and Queen round the wards: Major-General Lee, chairman of the Board of Management; Lieut.-Colonel Bruce Vaughan, chairman of the House Committee; Dr. Herbert Vachell, senior physician; Mr. P. Rhys Griffiths, senior surgeon; Dr. C. W. Shepherd, resident medical officer; Miss E. A. Mont Wilson, matron; and Mr. Leonard D. Rea, secretary.

Although it was their Majesties' expressed wish that the visit was to be regarded as of a purely private and informal character, permission was graciously given for the Committee and Governors to witness the arrival of their Majesties at the hospital. For this purpose accommodation was provided by stands on either side of the main entrance, so that their Majesties were enthusiastically greeted upon arrival by prominent supporters of the institution, including about 150 working men and colliers' representatives.

The exterior of the hospital buildings was gracefully decorated, while the path leading from the street to the vestibule was covered by an awning; but, inside, the beauty of the wards and green-tiled corridors was more fully revealed by their normal condition, save for a display of flowers and plants—of more than customary brilliance—conspicuous amongst which were bouquets sent by the Queen from the Royal yacht on the previous evening.

THE KING'S INTEREST IN MURAL DECORATION.

As time would not permit all the wards to be visited, a selection was made which comprised every class of patients, and no fewer than 130 sick people received happiness and encouragement from their Majesties' gracious words of friendly solicitude. The first ward visited was "The Coronation" Ward, which commemorates the Coronation of their Majesties and the Investiture of the Prince of Wales at Carnarvon. These historic events are illustrated by picture tiles, which elicited warm encomiums from their Majesties. The Queen was graciously pleased to accept at the hands of Colonel Bruce Vaughan a series of books, containing photographs and explanatory letterpress of the picture tiles in this and other wards.

The interest in the pictures, however, was not so great as their Majesties' interest in the patients, some of whom were tiny children, with the unselfconsciousness of childhood, each wearing a medal given by the Lord Mayor to mark the occasion.

Their Majesties thought of everyone. Nobody was forgotten. A gentle word here, a bright smile there, a touch of the hand where voice or eye would fail to reach, but the message was always there and the children knew it. "How do you do, Queen?" said one little girl, and her Majesty was delighted by the greeting—which is not so unconventional in children's wards as it would be at Windsor or Buckingham Palace.

THE JOHN NIXON WARD, KING EDWARD VII.'S HOSPITAL, CARDIFF, VISITED BY THE KING AND QUEEN.

So reluctant was the Queen to leave this ward that it was feared a stay here would not permit the completion of the tour, but all in good time their Majesties proceeded along the balcony, with words of gracious commendation for the beauty of the fountain garden below, and entered the "John Nixon" Ward, of which we publish an illustration. It also accommodates children, and is decorated by picture tiles illustrative of well-known nursery rhymes. Here Mrs. John Nixon, the donor of the ward, had the honour of being presented to their Majesties, who expressed the warmest appreciation of Mrs. Nixon's great generosity and of the form of its expression.

The "St. David's" Ward, with its happy reminiscence of the great Bute family, next engaged the attention of the King and Queen, who were then conducted upstairs to the "Llanbradach," "W. James Thomas," and "Thomas Webb" Wards, all of which accommodated men surgical cases.

Their Majesties' keen interest was more than maintained when, in the "W. James Thomas" Ward, Mr. W. James Thomas, the donor of the ward and the most substantial benefactor of the hospital, had the honour of being presented. As a matter of fact, the Queen did not wait for a formal presentation, but upon Mr. Thomas's presence being indicated, her Majesty greeted him with expressions of delight at the beauty of the ward—which also is decorated with picture tiles, illustrating events in Welsh history, especially those of local significance —and both the King and the Queen spoke in terms of high admiration of Mr. Thomas's well-directed generosity.

The William James Thomas Ward, King Edward VII.'s Hospital, Cardiff.

The remaining wards visited were the "Bute" and "Thompson" Wards, situated in the old building. The women patients gave their Majesties a welcome of quite Celtic fervour, and time was left for only a passing glance at the beautiful "Lady Aberdare" operating theatre, which, in common with the "Thompson" Ward, testifies to Lady Aberdare's consistent and specially personal interest in the Cardiff Hospital. During the course of the visit their Majesties frequently commented on the attractive appearance of the hospital, and even remarked that they had never seen brighter wards.

### The King and the Night Porter.

As illustrating his Majesty's keenness for observing every detail and circumstance, it may be related that upon leaving the "Bute" Ward his Majesty identified as an old Royal Marine, Mr. J. T. Brown, the night porter of the institution, who was stationed in the corridor, wearing his Service medal. His Majesty made kindly inquiries of Mr. Brown as to how long he had been in the Service and when he left.

Their Majesties left the institution by way of the out-patient department, the great hall of which provided additional accommodation for a number of Governors of the institution, but before leaving both the King and Queen inaugurated the visitors' book, which marks the new era upon which the institution has entered, with its new name of King Edward VII.'s Hospital, Cardiff, the assumption of which had previously been permitted by King George.

A few minutes after their Majesties' departure a special messenger arrived with a huge bunch of roses and a box of carnations, the personal gifts of the Queen. These were thoughtfully distributed by the Matron amongst the patients who did not occupy the wards through which their Majesties passed.

The conversations which the various officials of the hospital were privileged to have with their Majesties will be treasured as precious memories. The Matron had the honour of being in attendance on her Majesty, and her services during the South African campaign were appreciatively recalled by the King; whilst Dr. Shepherd, the resident medical officer, was in constant requisition to impart, for the Royal visitors' information, particulars relating to the various patients.

In going through the out-patient hall the Secretary, Mr. Rea, was honoured by inquiries from his Majesty with reference to the administration of the hospital. Mr. Rea, after supplying the information, remarked that the visit would prove an inspiration to hospital work in the Principality.

The truth of this had indeed already been fully proved by a letter received by Lieut.-Colonel Bruce Vaughan from an anonymous friend, who promised to contribute ten thousand guineas towards the building of the new physiological block in connection with the medical school, as an expression of his gratitude to their Majesties for their gracious visit.

The warmest congratulations of all interested in hospital development will be extended to that devoted pioneer of this great work in South Wales, Lieut.-Colonel Bruce Vaughan, whose work and foresight, extending over ten years, have done so much to convert a relatively small and ill-equipped hospital into one of first-class importance, qualifying, as it now does, for the establishment of a medical school in connection with the University of Wales.

## Figures and Tables

**Figure f1:**
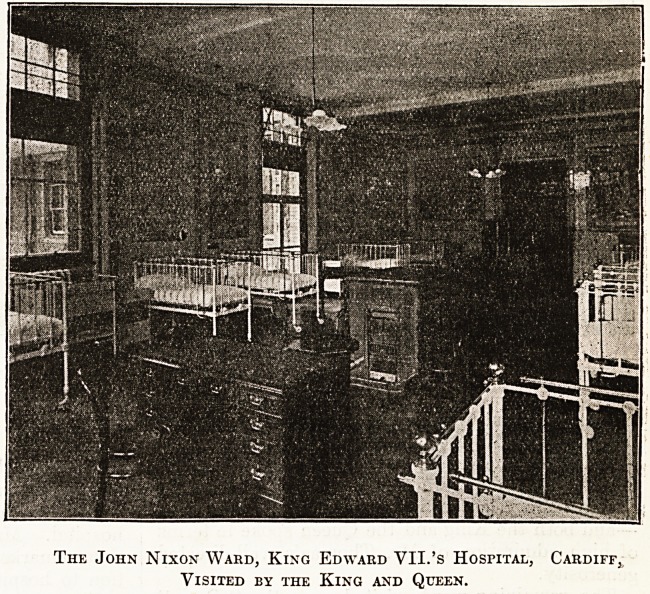


**Figure f2:**